# Oncogenic long noncoding RNA landscape in breast cancer

**DOI:** 10.1186/s12943-017-0696-6

**Published:** 2017-07-24

**Authors:** Shouping Xu, Dejia Kong, Qianlin Chen, Yanyan Ping, Da Pang

**Affiliations:** 1 Department of Breast Surgery, Harbin Medical University Cancer Hospital, 150 Haping Road, Harbin, 150081 China; 2 College of Bioinformatics Science and Technology, Harbin Medical University, Harbin, China; 3 Heilongjiang Academy of Medical Sciences, 157 Baojian Road, Harbin, 150086 China

**Keywords:** LncRNAs, Prognosis, Tumourigenesis, Breast cancer, TINCR

## Abstract

**Background:**

Few long noncoding RNAs (lncRNAs) that act as oncogenic genes in breast cancer have been identified.

**Methods:**

Oncogenic lncRNAs associated with tumourigenesis and worse survival outcomes were examined and validated in Gene Expression Omnibus (GEO) and The Cancer Genome Atlas (TCGA), respectively. Then, the potential biological functions and expression regulation of these lncRNAs were studied via bioinformatics and genome data analysis. Moreover, progressive breast cancer subtype-specific lncRNAs were investigated via high-throughput sequencing in our cohort and TCGA validation. To elucidate the mechanisms of the regulation of these lncRNAs, genomic alterations from the TCGA, Broad, Sanger and BCCRC data, as well as epigenetic modifications from GEO data, were then applied and examined to meet this objective. Finally, cell proliferation assays, flow cytometry analyses and TUNEL assays were applied to validate the oncogenic roles of these lncRNAs in vitro.

**Results:**

A cluster of oncogenic lncRNAs that was upregulated in breast cancer tissue and was associated with worse survival outcomes was identified. These oncogenic lncRNAs are involved in regulating immune system activation and the TGF-beta and Jak-STAT signalling pathways. Moreover, TINCR, LINC00511, and PPP1R26-AS1 were identified as subtype-specific lncRNAs associated with HER-2, triple-negative and luminal B subtypes of breast cancer, respectively. The up-regulation of these oncogenic lncRNAs is mainly caused by gene amplification in the genome in breast cancer and other solid tumours. Finally, the knockdown of TINCR, DSCAM-AS1 or HOTAIR inhibited breast cancer cell proliferation, increased apoptosis and inhibited cell cycle progression in vitro.

**Conclusions:**

These findings enhance the landscape of known oncogenic lncRNAs in breast cancer and provide insights into their roles. This understanding may potentially aid in the comprehensive management of breast cancer.

**Electronic supplementary material:**

The online version of this article (doi:10.1186/s12943-017-0696-6) contains supplementary material, which is available to authorized users.

## Background

Although great advances have been made in the management of breast cancer over the past decade, patient outcomes still merit consideration due to the high rate of tumour-specific death [1, 2]. The molecular mechanisms of tumourigenesis are still unclear in breast cancer. Thus, identifying new genes related to tumourigenesis and patient prognosis, as well as elucidating the molecular mechanisms underlying these oncogenic processes, are urgently required.

It is certified that less than 2% of the genome encodes proteins, but at least 75% are transcribed into noncoding RNAs [3]. Generally, long noncoding RNAs (lncRNAs) are defined as transcripts that are longer than 200 nucleotides and have no protein-coding capacity [4]. There are more than 120,000 transcripts annotated as lncRNAs in the human genome as released by the Encyclopedia of DNA Elements (ENCODE) Project Consortium (release 28) [5]. LncRNAs share common features with mRNAs: many of them are transcribed by RNA polymerase II and undergo 5′-capping, polyadenylation and splicing [3]. In addition, the histone profiles of lncRNAs are similar to protein-coding genes for the active histone markers H3K4me2, H3K4me3, H3K9ac and H3K27ac [6]. On the other hand, lncRNAs have several distinct features that distinguish them from protein-coding mRNAs. Generally, lncRNAs lack coding potential due to fewer exons [7]. However, there are some exceptions with the development of lncRNA investigations. LINC00961 regulates mTORC1 and muscle regeneration by encoding SPAR polypeptide [8]. Functional polypeptides of myoregulin encoded by LINC00948 and Dworf encoded by NONMMUG026737 have been reported to modulate SERCA pump activity [9, 10]. LncRNA CRNDE encodes a nuclear peptide, CRNDEP, which is overexpressed in highly proliferating tissues and is involved in cell turnover [11]. Moreover, many investigations have shown that lncRNAs are of relatively lower expression levels than protein-coding genes; however, they exhibit a more cell type-specific pattern [12–14]. Unlike mRNAs, most lncRNAs are localized to the nucleus, while mRNA is in the cytoplasm [6]. Finally, lncRNAs belong to evolutionary conserved families evolving faster than mRNAs, where sequence similarity is likely to be preserved mainly in regions with secondary structure formation [6, 12].

LncRNAs play an important role in regulating gene expression at various levels, including alternative splicing, regulation of protein activity, and alteration of protein localization, as well as chromatin modification, transcription, and posttranscriptional processing [3, 15–21]. The mechanisms by which lncRNAs contribute to the regulatory networks that underpin cancer development are diverse [22, 23]. Unlike microRNA or piwi-interacting RNA (piRNA), lncRNAs drive many important cancer phenotypes through their interactions with other cellular macromolecules such as DNA, protein, and RNA [22–26]. Regarding their role in malignancy, lncRNAs are associated with immortal-associated characteristics, including cell cycle regulation, survival, immune response or pluripotency in cancer cells [27–32]. Several common lncRNAs have been investigated in cancers. For example, HOTAIR promotes cancer metastasis by reprogramming the chromatin state in a manner dependent on PRC2 [33]. SChLAP1 contributes to the development of lethal cancer by antagonizing the tumour-suppressive functions of the SWI/SNF complex [34]. Another lncRNA, SAMMSON, increases the pro-oncogenic function via interacting with p32 in melanoma [35]. Despite growing knowledge regarding the molecular mechanisms of lncRNA functions in malignancy, the modes of action of most lncRNAs in breast cancer remain unclear. Aberrant expression of oncogenic lncRNAs may confer capacities for tumour initiation, growth, and metastasis in breast cancer cells, thus leading to a worse prognosis for patients. Similarly, applying antisense oligonucleotides can inhibit the expression of the candidate therapeutic target Malat1 successfully in an MMTV-PyMT mouse model with breast cancer [36]. Thus, unravelling the landscape of these oncogenic lncRNAs in breast cancer is essential and urgent.

The aim of this study was to identify the predictive capability of oncogenic lncRNAs for the tumourigenesis and prognosis of breast cancer. In this study, 1088 breast cancer patients from GEO data were selected and analysed to investigate the lncRNAs associated with tumourigenesis and outcomes. The aberrant long noncoding RNAs were then validated using TCGA data and high-throughput sequencing in our cohort. Genome-wide in silico analysis and in vitro assays revealed the potential biological functions of these lncRNAs, including their mechanisms of regulation and roles in tumourigenesis. Moreover, identification and validation of breast cancer subtype-specific lncRNAs and the determination of their association with clinical outcomes were also investigated.

## Methods

### Public data access and analysis

GEO data (GSE21653, GSE31448, GSE10810, GSE29431, GSE23177, GSE42568, and GSE48391) were downloaded and processed (http://www.ncbi.nlm.nih.gov/geo/). The genome-wide lncRNA expression profiles for breast cancer, renal cancer, and lung cancer were downloaded from TCGA (https://tcga-data.nci.nih.gov/). For the microarray analysis, we adjusted the signal values for low-abundance genes. A signal value lower than log3 was set to log3. Moreover, the invariant genes (i.e., same expression value across all samples) and low-variation genes were filtered. Genes that were detected in less than 50% of the profiled samples were also filtered. The SAM method was applied, and we implemented a series of steps to estimate the significance of difference and false discovery rate for each filtered gene as previously described [37]. A method to estimate the significance of difference and false discovery rate for each filtered gene was implemented as follows:

1. Calculate the exchange factor s_0_. First, we calculated the standard deviation of geneexpression for all genes s_*i*_, denoted s^*α*^, as the α percentile for s_*i*_. The relative difference in gene expression (d Score) at the α percentile is calculated as ${d}_i^{\alpha }={r}_i/\left({s}_i+{\mathrm{s}}^{\alpha}\right)$, where *r*_*i*_ is the fold change of gene expression for gene i between two conditions. Next, each interval of the percentile value q_1_ < q_2_ <  ···  < q_100_ of the s_*i*_ and the mean absolute deviation of ${d}_i^{\alpha }{\mathrm{v}}_j= mad\left\{{d}_i^{\alpha }={r}_i/\left({s}_i+{\mathrm{s}}^{\alpha}\right)|{s}_i\in \Big[{q}_j,{q}_{j+1}\Big)\right\}$ were calculated. Finally, we selected the α (denote as $\widehat{\alpha}$) to make the CV (coefficient of variation) of the v_*j*_ achieve a minimum, and set the exchange factor ${\mathrm{s}}_0={\mathrm{s}}^{\widehat{\alpha}}$.

(2) Calculate the statistical value (d Score) for every gene i:

*d*_*i*_ = *r*_*i*_/(*s*_*i*_ + s_0_),

(3) Calculate the order statistic according to *d*_*i*_:
*d*_(1)_ ≤ *d*_(2)_ ≤  ···  ≤ *d*_(*i*)_ ≤  ···  ≤ *d*_(*p*)_,
(4) Perform 1000 permutations to estimate the expected distribution of the d Score. We denote the estimated statistical values:
$$ {d}_{(1)}^{\ast}\le {d}_{(2)}^{\ast}\le \dots \le {d}_{(i)}^{\ast}\le \dots \le {d}_{(p)}^{\ast } $$,
(5) Obtain the order statistic value under the permutation:
$$ {\overset{-}{d}}_{(i)}=\frac{\sum_{i=1}^{1000}{d}_{(i)}^{\ast }}{1000} $$,
(6) By calculating the maximum distance between the order statistic *d*_(*i*)_ and the expected order statistic ${\overset{-}{d}}_{(i)}$, we would construct a series of reject regions for the q-value.
(7) For a fixed delta value, we computed the difference ${\Delta}_{(i)}={d}_{(i)}-{\overset{-}{d}}_{(i)}$ and found the nearest Δ_(*i*)_ for gene i. The cut-up was marked as min{Δ_(*i*)_ ≥ delta} for the positive gene and the cut-down as max{Δ_(*i*)_ ≤  − delta} as for negative gene. Next, we called these genes significantly positive genes, whose difference was larger than the cut-up value, and significantly negative genes, whose difference was smaller than the cut-down value.
(8) Estimate the false discovery rate (FDR):
$FDR=\frac{V_{(p)}}{R_{(p)}}$, where *V*_(*p*)_ *i*s the number of positive genes called in the 1000 permutations, and *R*_(*p*)_ is the median of the number of false-positive genes for the above permutations.
(9) Obtain the q-value for gene i by selecting the minimum of the FDR for the 50 delta values determined in step (7).

Welch’s t-test (unequal variances) and analysis of variance were also applied for two-group and multiple-group analyses, respectively. For multiple-comparison analysis, the q-value was used to control the false discovery rate. Clustering heatmap analysis was performed according to previous study [38].

### Guilt-by-association analysis

To identify a list of lncRNAs positively and negatively correlated with the target genes, data from the TCGA were evaluated to compute a pairwise Pearson correlation between the expression of the target lncRNA and all the genes. Only associated genes with an absolute *r* ≥ 0.4 and a significant correlation (*P* < 0.05) were retained. Gene ontology term enrichment (GO) and Kyoto Encyclopedia of Genes and Genomes (KEGG) pathway analysis were analysed using DAVID as previously described [39, 40].

### Patients and clinical samples

Thirty invasive breast cancer and adjacent non-cancerous tissues were obtained from patients who had not received either chemotherapy or radiotherapy and who were treated at the Department of Breast Surgery at Harbin Medical University Cancer Hospital in 2014. This study was approved by the research ethics committee of the Harbin Medical University Cancer Hospital. Written informed consent was obtained from all the patients who participated in the study. The clinicopathological characteristics of the patients are presented in Additional file 1: Table S1.

### Library preparation for lncRNA sequencing

A total of 3 μg of RNA per sample was used for downstream RNA sample preparations. Ribosomal RNA was removed using the Ribo-Zero™ Gold kit (Epicentre, Wisconsin, USA). Subsequently, sequencing libraries were generated according to the manufacturer’s recommendations. The libraries were sequenced on an Illumina HiSeq 2500 platform, and 100-bp paired-end reads were generated. Raw sequencing and processed RNA-Seq data of this study have been deposited to the NCBI Gene Expression Omnibus database under accession number GSE71651 (http://www.ncbi.nlm.nih.gov/geo/query/acc.cgi? token = obcxosaur xoppwx & acc = GSE71651).

### Cell culture experiments

MDA-MB-453 and MCF-7 cells were cultured in DMEM (Invitrogen, Carlsbad, CA) containing 10% foetal bovine serum and 100 units/ml penicillin/streptomycin at 37 °C in an atmosphere containing 5% CO_2_. UACC812 and T47D cells were cultured in 1640 medium (Invitrogen, Carlsbad, CA) containing 10% foetal bovine serum and 100 units/ml penicillin/streptomycin at 37 °C in an atmosphere containing 5% CO_2_. All the cell lines were obtained from the Chinese Type Culture Collection, Chinese Academy of Sciences. Cells were used during their logarithmic growth phase.

### Small interfering RNA (siRNA) and qRT-PCR

Breast cells were grown in complete medium before transfection with siRNAs using Lipofectamine 2000 (Life Technologies, 11,668–019) according to the manufacturer’s protocol. Two siRNAs each were designed to target TINCR (siR_1:5′- GCAUGAAGUAGCAGGUAUUUU-3′ and siR_2: 5′-GAUCCCGAGUGAGUCAGAA UU-3′), HOTAIR (siR_1: 5′-CCACAUGAACGCCCAGAGAUU-3′ and siR_2: 5′-GAACGGGAGUACAGAGAGAUU-3′) and DSCAM-AS1 (siR_1: 5′- ACUCAUCCAUGUACCCAUUUCUUAA-3′ and siR_2: 5′-CCUCCUCCAACUGCCAUU UAUUUAU-3′). qRT-PCR analysis was performed using the SYBR-Green method, and the specific sequences of the primers used were as follows: 5′-TGTGGCCCAAACTCAGGGATACAT-3′ (forward) and 5′-AGATGACAGTGGCTGGAGTTGTCA-3′(reverse) for TINCR, 5′- GTCCCTAATATCCCGGAGGT-3′ (forward) and 5′-GCAGGCTTCTAAATCCGTTC-3′ (reverse) for HOTAIR; 5′-GATCCTTGTTTGGTCTCACTCC-3′(forward) and 5′-ATGCCTATGTGGGTGATTGG-3′(reverse) for DSCAM-AS1; and 5′-TTTGATGGTGACCTGGGAAT-3′ (forward) and 5′-GAACATCTGGCTGGTTCACA-3′ (reverse) for ERBB2; 5′-ACCACAGTCCATGCCATCAC-3′ (forward) and 5′- TCCACCCTGTTGCTGTA-3′ (reverse) for GAPDH. Primers for MiR-125b and U6 were obtained as previously described [41]. Quantitative normalization of target cDNA was performed for each sample using GAPDH/U6 expression as an internal control. The relative levels of TINCR, HOTAIR, DSCAM-AS1, ERBB2, MiR-125b vs. GAPDH/U6 were determined by the comparative CT (2^−ΔΔCT^) method.

### Cell proliferation assays

Cell proliferation assays were performed using the Cell Counting Kit-8 according to the manufacturer’s instructions (Beyotime, Shanghai, China). Briefly, 2 × 10^3^ cells were seeded in a 96-well plate. Cell proliferation was assessed at 24, 48, and 72 h. After the addition of 20 μl of WST-1 reagents per well, cultures were incubated for 2 h, and the absorbance was measured at 450 nm using a microplate reader (BioTek, VT, United States).

### Flow cytometry

An Annexin-PE Apoptosis detection kit (BD Biosciences, San Jose, CA) was used to examine cell apoptosis according to the manufacturer’s instructions. Briefly, cells were washed twice in cold PBS and then harvested and resuspended in 1× binding buffer. Next, 100 μl of the cell solution (1 × 10^5^ cells) was transferred to a 5-ml culture tube, and 5 μl of annexin V-PE and 5 μl of 7-AAD were added into culture tube. The cells were gently vortexed and incubated for 15 min at RT (25 °C) in the dark. Next, 400 μl of 1× binding buffer was added to each tube, and apoptosis analysis was performed using a FACScan instrument (Becton Dickinson, Mountain View, CA, USA). For cell cycle analysis, the CycleTEST™ PLUS DNA Reagent Kit (BD. Cat No.340242) was used, and the experiment was performed as previously described [42].

### TUNEL assays

Apoptosis-induced DNA fragmentation was performed using the transfers-mediated deoxyuridine triphosphate (dUTP)-digoxigenin nick end-labelling (TUNEL) assay. UACC812 and MDA-MB-453 cells were plated in 24-well flat-bottomed plates at a density of 1 × 10^5^ cells per well, and cells were fixed in 4% (*w*/*v*) paraformaldehyde at 4 °C for 25 min. TUNEL staining was examined using the in situ cell death detection kit (Roche), and the nuclei were stained with DAPI for 10 min according to the manufacturer’s instructions. The numbers of TUNEL-positive cells were captured with a fluorescence microscope (Olympus), and the ratio of apoptosis cells was determined with ImagePro Plus software.

### Statistical analyses

The times of OS and RFS were calculated as the time from surgery until the occurrence of death or relapse, respectively. The expression of lncRNA was dichotomized using a study-specific median expression as the cut-off to define “high value” at or above the median versus “low value” below the median. The differences between the groups in our in vitro experiments were analysed using Student’s t-test. Spearman correlation coefficients were calculated for correlation analysis. All the experiments were performed in triplicate, and SPSS 16.0 software (SPSS, Chicago, IL) was used for statistical analysis. All statistical tests were two-sided, and *P* < 0.05 was considered to be statistically significant.

## Results

### Aberrant gene expression landscape in breast cancer

**Fig. 1:**
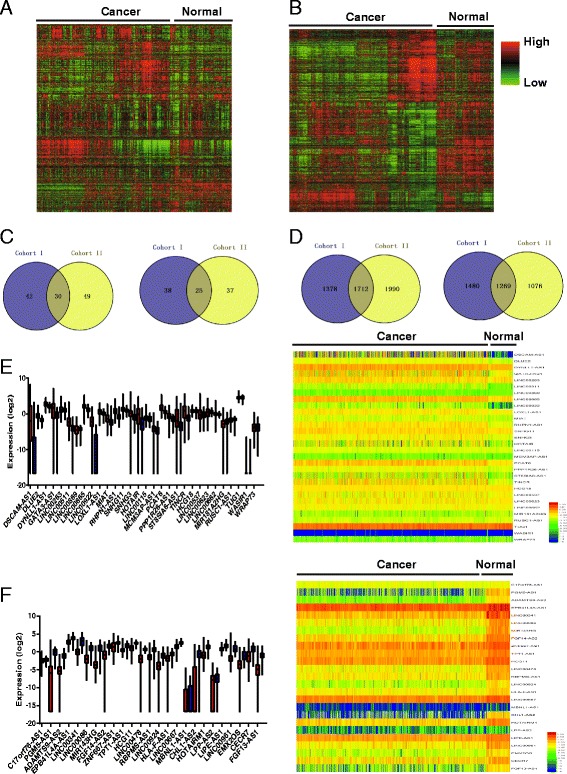
Aberrant gene expression profile in breast cancer. **a** Hierarchical clustering of differentially expressed genes in breast cancer relative to normal tissue in cohort I (*N* = 623). **b** Hierarchical clustering of differentially expressed genes in breast cancer relative to normal tissue in cohort II (*N* = 465). Red through blue colour indicates high to low expression levels, respectively. **c** Upregulated (*left*) and downregulated (*right*) lncRNAs from cohort I and cohort II identified via Venny online software analysis (http://bioinfogp.cnb.csic.es/tools/venny/). **d** Upregulated (*left*) and downregulated coding genes (*right*) from cohort I and cohort II. **e** Expression levels of 30 upregulated lncRNAs in cancer relative to normal tissue (*left*) and hierarchical clustering of these genes (*right*) in the TCGA. **f** Expression levels of 25 downregulated lncRNAs in cancer relative to normal tissue (*left*) and hierarchical clustering of these genes (*right*) in the TCGA. The *red columns* indicate the expression levels of lncRNAs in cancer tissues, and the blue columns represent normal tissues

Two cohorts containing seven datasets, including 1088 breast cancer patients, were selected from GEO data and were analysed in this study. In cohort I (GSE21653 and GSE31448), 137 lncRNAs and 8914 coding genes showed significantly aberrant expression between breast cancer and normal tissue (*P* < 0.05); however, in cohort II (GSE10810, GSE29431, GSE23177, GSE42568, and GSE48391), 164 lncRNAs and 9685 coding genes were differentially expressed in breast cancer and normal tissues (*P* < 0.05) (Fig. 1a, b and Additional file 2: Table S2 and Additional file 3: Table S3). Next, the overlapping genes were compared across these two cohorts. There were 30 and 25 lncRNAs upregulated and downregulated, respectively, in both of these cohorts (*P* < 0.05) (Fig. 1c and Additional file 2: Table S2). For the coding genes, there were 1712 and 1269 mRNAs upregulated and downregulated, respectively (*P* < 0.05) (Fig. 1d and Additional file 3: Table S3). To further confirm the result in a third independent cohort, the aberrant lncRNAs were validated using TCGA data. As expected, all except one lncRNA (WASIRS1) showed similar expression profiles as those from the in silico analysis (Fig. 1e, f).

### Potential biological functions of aberrantly expressed lncRNAs in breast cancer

**Fig. 2:**
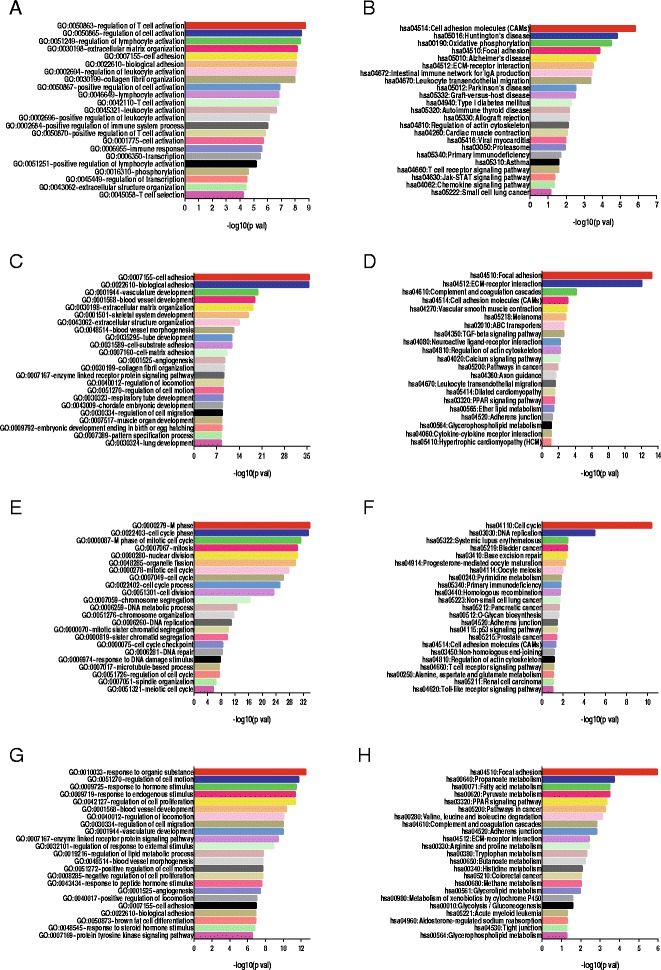
Potential biological function of aberrantly expressed lncRNAs in breast cancer. **a** & **b** Gene ontology enrichment analysis (*left*) and KEGG analysis (*right*) for 30 upregulated lncRNAs from cohort I and cohort II. **c** & **d** Gene ontology enrichment analysis (*left*) and KEGG analysis (*right*) for 25 downregulated lncRNAs from cohort I and cohort II. **e** & **f** Gene ontology enrichment analysis for 1712 upregulated coding genes (*left*) and 1269 downregulated coding genes (*right*) from cohort I and cohort II. **g** & **h** KEGG analysis for 1712 upregulated coding genes (*left*) and 1269 downregulated coding genes (*right*) from cohort I and cohort II. *P*-values <0.05 were defined as statistically significant. The *vertical axis* represents the biological procession or pathway category, and the *horizontal axis* represents the –log10 (*P* value) of these significant biological processions or pathways

To explore the possible functions of these lncRNAs, guilt-by-association analyses were used, as described in the Methods section. GO and KEGG pathway analysis were also investigated for the identified lncRNAs. GOTERM_BP_DIRECT was selected to investigate the related biological process of enriched genes. The main related functions of the upregulated lncRNAs were regulation of lymphocyte activation, extracellular matrix organization, and cell adhesion, and the main pathways or proteins that were involved included cell adhesion molecules, the T-cell receptor signalling pathway, ECM-receptor interaction and the Jak-STAT signalling pathway (Fig. 2a, b and Additional file 4: Table S4). The results of GOTERM_CC_DIRECT and GOTERM_MF_DIRECT for upregulated lncRNAs were also performed (Additional file 5: Table S5). For the downregulated lncRNAs, cell adhesion, extracellular matrix organization, extracellular structure organization and angiogenesis were identified in the GO function analysis, and focal adhesion, ECM-receptor interaction, ABC transporter activity and the TGF-beta signalling pathway were identified in the KEGG pathway analysis (Fig. 2c, d and Additional file 4: Table S4). Moreover, GO and KEGG analyses were performed for the coding genes. The results indicated that the upregulated genes were associated with cell cycle phase and mitosis, whereas the downregulated genes were associated with the regulation of cell motility, cell proliferation, cell adhesion, and angiogenesis (Fig. 2e, f and Additional file 6: Table S6). For KEGG analysis, cell cycle, DNA replication, the p53 signalling pathway, and the Toll-like receptor signalling pathway were identified for the upregulated genes, and focal adhesion, the PPAR signalling pathway, ECM-receptor interaction and glycolysis/gluconeogenesis were identified for the downregulated genes (Fig. 2g, h and Additional file 6: Table S6).

### Oncogenic lncRNAs associated with progressive breast cancer

**Fig. 3:**
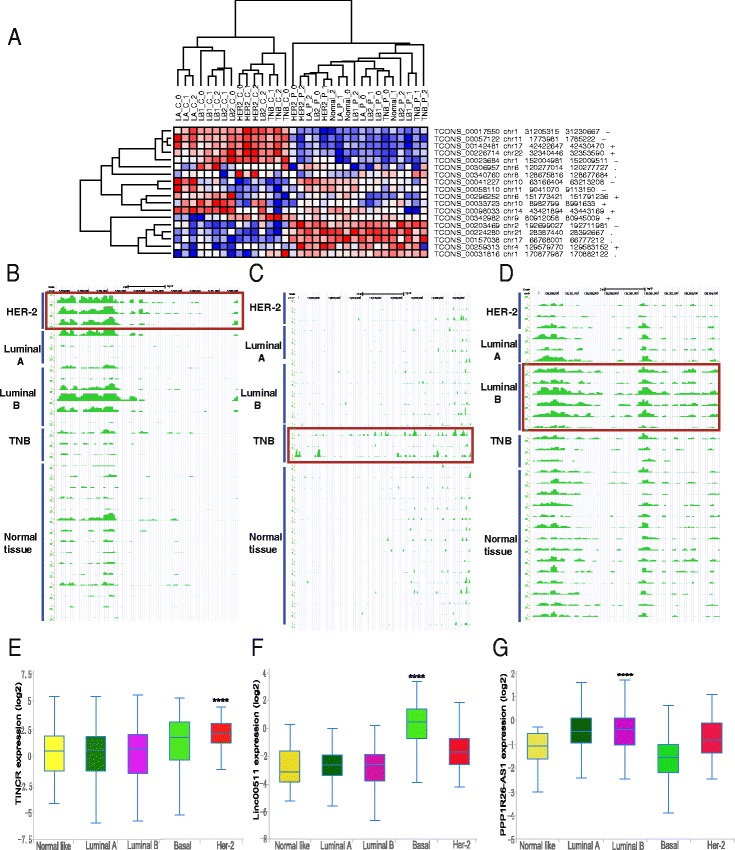
Progressive breast cancer subtype-specific lncRNA identification and validation. **a** Hierarchical clustering of differentially expressed genes in breast cancer tissues (*N* = 15), adjacent normal tissues (*N* = 15) and normal breast tissue from non-cancer patients (*N* = 3) via RNA-Seq performed by our research group. Red through blue colour indicates high to low expression levels, respectively. **b**, **c** and **d** RNA-Seq data visualization of TINCR (**b**) and Linc00511 (**c**) and PPP1R26-AS1 (**d**) representing HER-2, triple-negative and luminal B subtype-specific lncRNAs, respectively. **e**, **f** and **g** Validation of TINCR (**e**), Linc00511 (**f**) and PPP1R26- AS1 (**g**) as progressive breast cancer subtype-specific lncRNAs in the TCGA. The expression level of each gene was measured by log2 FPKM. **** *P* < 0. 0001

Breast cancer is highly heterogeneous and can be divided into the following five subclasses: luminal A, luminal B (HER-2 positive), luminal B (HER-2 negative), HER-2 subtype and triple negative [1]. Owing to the aggressive nature of the triple-negative and HER-2 subtypes, we wondered whether aggressive breast cancer subtype-specific lncRNAs (BCSPLs) might exist. Thus, 33 specimens, including each type of breast cancer tissue (*N* = 15) and adjacent normal tissues (*N* = 15), as well as breast tissue from non-cancer patients (*N* = 3), were obtained and subjected to RNA-Seq analysis performed by our research group (Fig. 3a and Additional file 7: Table S7). Among the 30 upregulated lncRNAs from the in silico analysis, three lncRNAs were confirmed to be subtype specific in our RNA-Seq results: TINCR, LINC00511, and PPP1R26-AS1 represented the HER-2, triple negative and luminal B subtypes, respectively (Fig. 3b–d). To avoid selection bias in the samples for BCSPL, TCGA data were used to validate these results. As expected, we obtained consistent results from the analysis of the public data (Fig. 3e–g).

Considering triple-negative breast cancer as one of the worst subtypes in breast cancer, LINC00511, as one of the triple-negative-subtype-specific lncRNAs, was selected for further analysis. By guilt-by-association analysis in the TCGA set, LINC00511 was associated with cell adhesion, cell migration, ERK1 and ERK2 cascade regulation and so on by GO analysis (Additional file 8: Table S8). Moreover, it was involved in glycosphingolipid biosynthesis, cell adhesion molecules, proteoglycans and the p53 signalling pathway by KEGG analysis (Additional file 8: Table S8). Next, LINC00511 expression was higher in patients with BRCA1 mutation than in those with wild-type expression (Additional file 9: Figure S1a). Moreover, this phenomenon was observed in patients with RB1 mutation or TP53 mutation (Additional file 9: Figure S1b, c). Finally, the StarBase v2.0 database was applied to examine the relationship of LINC00511 and tumour suppressor microRNAs [43]. In this process, LINC00511 had binding sites of miR-29, miR-16, miR-195 and miR-497, respectively (Additional file 10: Table S9). Meanwhile, these microRNAs were reported to be tumour suppressors [44–47]. Thus, LINC00511 may be a bona fide therapy target in triple-negative breast cancer.

### Oncogenic lncRNAs are correlated with worse survival outcomes

**Fig. 4:**
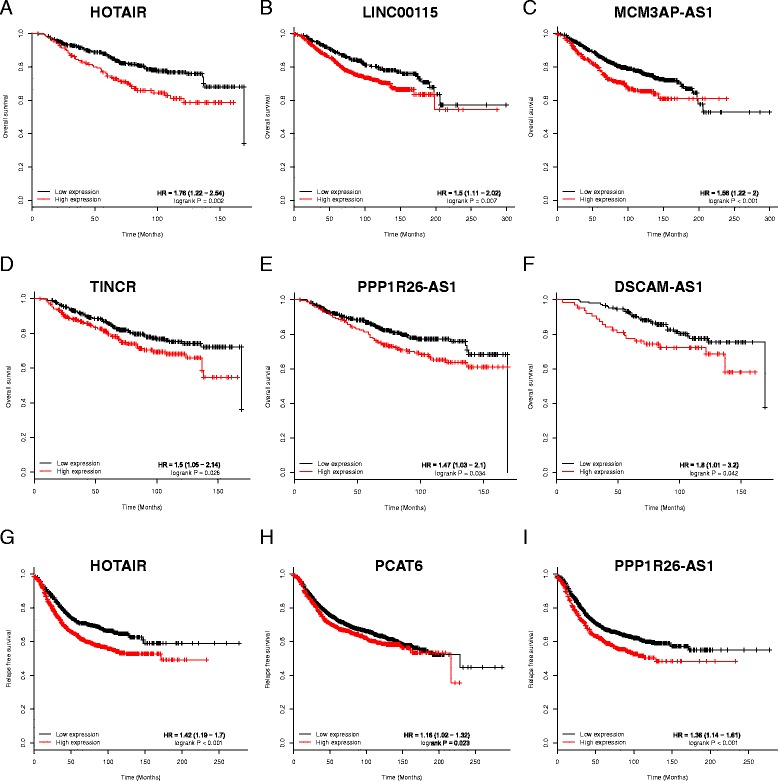
Upregulated lncRNAs and clinical outcomes. Overall survival rates of breast cancer patients compared with the levels of HOTAIR (**a**), LINC00115 (**b**), MCM3AP-AS1 (**c**), TINCR (**d**), PPP1R26-AS1 (**e**), and DSCAM-AS1 (**f**) in 26 datasets from GEO. Relapse-free survival of breast cancer patients compared with the levels of HOTAIR (**g**), PCAT6 (**h**) and PPP1R26-AS1 (**i**) in 26 datasets from GEO (*N* = 4140)

Next, we investigated the relationship between breast cancer prognosis and the 30 aberrant upregulated lncRNAs obtained via in silico analysis from cohorts I & II. GEO was rechecked to identify datasets that included all such genes and the patients’ long-term follow-up information. Finally, 26 datasets with 4140 breast cancer patients met these criteria and were used to identify breast cancer prognosis-associated lncRNAs (BCPALs) (Additional file 11: Table S10). Among these lncRNAs, HOTAIR, LINC00115, MCM3AP-AS1, TINCR, PPP1R26-AS1, and DSCAM-AS1 were confirmed to be BCPALs via log-rank overall survival analysis (Fig. 4a–f). In addition to overall survival, increased expression of HOTAIR, MCM3AP-AS1, and PCAT6 indicated worse relapse-free survival in these patients (Fig. 4g–i). Among the six BCPALs, only the increased expression of HOTAIR was associated with a worse prognosis; the remaining five BCPALs were first reported in this study.

### Regulation of the oncogenic lncRNAs

**Fig. 5:**
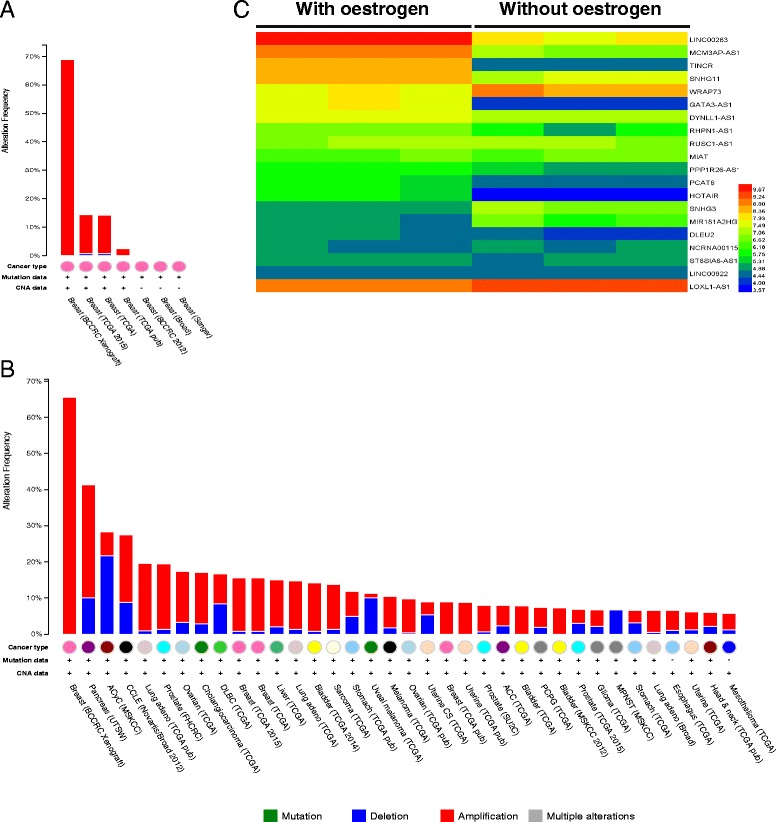
Regulation of the oncogenic lncRNAs. **a** Gene amplification was the main cause of the upregulation of these genes in breast cancer and other cancers, particularly ovarian cancer, bladder cancer and uterine cancer, based on the data from the TCGA, Broad, Sanger and BCCRC databases (**c**). **b** Hierarchical clustering of aberrantly expressed lncRNAs in breast cancer with (GSM678805, GSM678806 and GSM678807) or without (GSM678802, GSM678803 and GSM678804) oestrogen stimulation. *Red through blue* colour indicates high to low expression levels, respectively

The mechanisms of the upregulation of 30 lncRNAs were next explored via a genomic approach. Mutation, deletion, amplification, and multiple alterations were investigated for these lncRNAs in the TCGA, Broad and Sanger data. The results showed that the upregulation of these 30 lncRNAs is mainly caused by gene amplification in breast cancer (Fig. 5a). Moreover, gene amplification may lead to the upregulation of these lncRNAs in pan-cancer, particularly ovarian cancer, bladder cancer and uterine cancer (Fig. 5b). Next, the potential upstream regulation of these lncRNAs via transcription factor and epigenetic modifications was next identified in breast cancer (http://www.ncbi.nlm.nih.gov/gds/). The results indicated that DYNLL1-AS1, GATA3-AS1, LINC00263, LINC00922, MIAT, RHPN1-AS1, SNHG11, HOTAIR, MCM3AP-AS1, PCAT6, PPP1R26-AS1 and TINCR are positively regulated by oestrogen, whereas other lncRNAs, including DLEU2, LINC00511, LOXL1-AS1, SNHG3, LINC00115, MIR181A2HG, and WRAP73, are negatively regulated by oestrogen (Fig. 5c). Moreover, WRAP73, LINC00115, and PCAT6 may be negatively regulated by the Kruppel-like zinc finger protein ZNF217, whereas LOXL1-AS1 and MIAT may be positively regulated by this protein (Additional file 12: Table S11). DNA methylation and other transcription factors, such as GATA3 and LIM-only protein 4, were found to be involved in the regulation of similar lncRNAs (Additional file 12: Table S11).

### Validation of oncogenic roles of lncRNAs

**Fig. 6:**
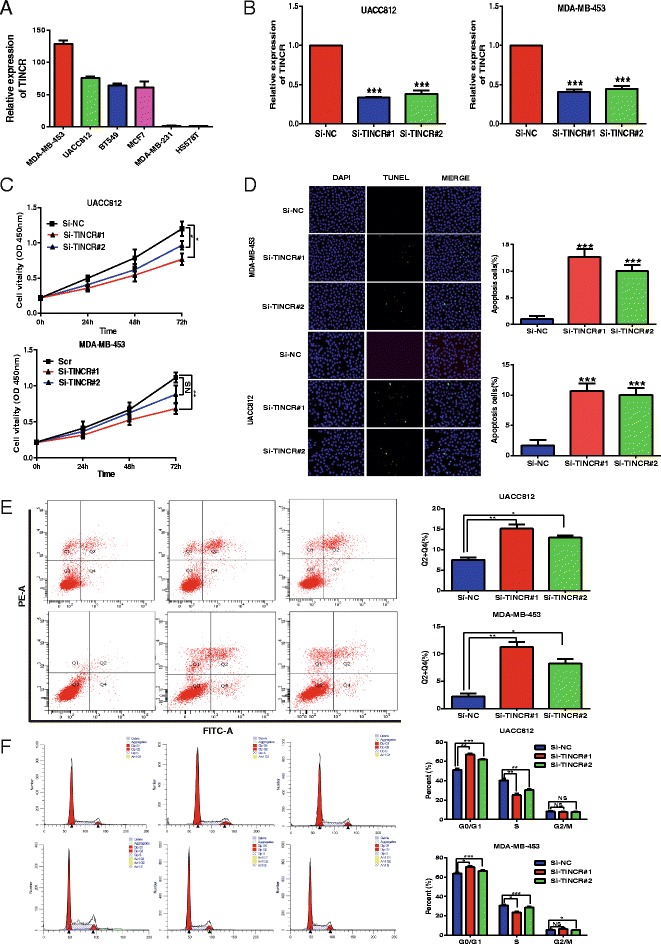
Oncogenic functions of TINCR. **a** The expression level of TINCR was examined in breast cancer lines. **b** Knockdown efficiency of siRNAs targeting TINCR in UACC812 and MDA-MB-453 cells, as determined by qRT-PCR. Quantitative normalization of TINCR was performed in each sample using GAPDH expression as an internal control. The relative levels of TINCR vs. GAPDH were determined by the comparative CT (2^−ΔΔCT^) method. **c** The CCK-8 assay was conducted to measure cell proliferation in UACC812 and MDA-MB-453 cells after the knockdown of TINCR. **d** TUNEL apoptosis analysis of UACC812 and MDA-MB-453 cells after the knockdown of TINCR. **e** Flow cytometry apoptosis analysis of UACC812 and MDA-MB-453 cells after the knockdown of TINCR. **f** Determination of the cell cycle distribution in UACC812 and MDA-MB-453 cells after the knockdown of TINCR via flow cytometry. *** *P* < 0.0 01;** *P* < 0. 01; * *P* < 0.05. Each assay was performed in triplicate

To interpret the potential biological functions of these lncRNAs in tumourigenesis, we chose TINCR, HOTAIR and DSCAM-AS1 for further biological function research because these lncRNAs showed the most significant differential expression in cohort I and cohort II. Due to the increased expression of such oncogenic lncRNAs being associated with worse outcomes, we sought to determine whether these lncRNAs might be involved in breast cancer tumourigenesis. The expression level of TINCR was examined in breast cancer lines (Fig. 6a). UACC812 and MDA-MB-453 cells were selected for the biological function study of TINCR. Two different siRNAs were designed and used to knock down TINCR. The knockdown efficiency was validated via qRT-PCR in vitro (Fig. 6b), and the CCK-8 assay results indicated that knockdown of TINCR inhibited UACC-812 or MDA-MB-453 cell proliferation, respectively (Fig. 6c). Moreover, increased apoptosis was observed after the downregulation of TINCR in UACC-812 and MDA-MB-453 cells via flow cytometry apoptosis detection and TUNEL assay, respectively (Fig. 6d, e). Finally, knockdown of TINCR, compared with controls, increased the percentage of the G0/G1 stage and decreased the percentage of S stage in UACC-812 and MDA-MB-453 cells (Fig. 6f).

Next, molecular interaction and potential oncogenic mechanism of TINCR were explored subsequently. Firstly, knockdown of TINCR increases the expression of Bax and decreases the expression of Bcl-2 in UACC-812 cell (Additional file 13: Figure S2a, b). This result was next validated using breast cancer data in TCGA (Additional file 13: Figure S2c, d). Secondly, co-expressed genes with TINCR were investigated (Additional file 14: Table S12) and cell proliferation function of these genes were detected via GO analysis (Additional file 13: Figure S2e). Finally, TargetScan database (release 3.1) was applied to predict mircroRNAs regulated by TINCR. MiR-125b was selected and validated in vitro using UACC-812 cell (Additional file 13: Figure S2f, g). It was reported that ERBB2 was a target of MiR-125b in solid cancers [41, 48, 49]. Thus, expression of MiR-125b and ERBB2 in breast cancer was investigated. As expected, MiR-125b was upregulated and ERBB2 was downregulated with TINCR knockdown (Additional file 13: Figure S2g, h). Taken together, TINCR promotes tumourigenesis partly via TINCR-MiR-125b-ERBB2 axis and its negative regulation of apoptosis pathway in breast cancer. For HOTAIR and DSCAM-AS1, such experiments to examine their oncogenic roles were carried out in breast cancer cell lines. As expected, the knockdown of DSCAM-AS1 or HOTAIR inhibits cell proliferation, promotes cell apoptosis, and increases the ratio of G0/G1 stage in MCF7 and T47D cells (Additional file 15: Figure S3a, b, c, d, e). Together, these results indicate that these lncRNAs may play oncogenic roles in the tumourigenesis of breast cancer.

## Discussion

Oncogenic lncRNAs in breast cancer were identified and validated comprehensively via genome-wide in silico analysis and biological experiments in this study. To our knowledge, only a few lncRNAs that regulate the process of tumourigenesis and that are associated with the prognosis in breast cancer have been identified. In this study, the identified lncRNAs were found to be associated with essential biological functions in cancer, including the regulation of immune system activation, cell adhesion, angiogenesis, ABC transporter activity, and TGF-beta and Jak-STAT signalling. Moreover, TINCR, LINC00511, and PPP1R26-AS1 were identified as HER-2, triple-negative and luminal B subtype-specific lncRNAs, respectively. In addition, BCPALs, including HOTAIR, LINC00115, MCM3AP-AS1, TINCR, PPP1R26-AS1, and DSCAM-AS1, were identified and confirmed via log-rank analysis. Next, gene amplification in the genome appeared to be the main underlying mechanisms for the upregulation of these oncogenic lncRNAs. Finally, the oncogenic roles of TINCR, HOTAIR and DSCAM-AS1, which showed the most significant differential expression in cohort I and cohort II, were selected and performed in vitro. Knockdown of each of the above lncRNA inhibited proliferation in breast cancer cell lines, increased apoptosis and inhibited cell cycle progression. Together, these results indicated that the lncRNAs identified and validated in this study play oncogenic roles in breast cancer.

Among the 30 aberrantly upregulated lncRNAs identified in this study, only 7 have previously been reported to be associated with malignancy, and the potential functions of the other 23 lncRNAs remain a mystery. Increased SNHG3 expression is associated with malignant status and worse overall survival, recurrence-free survival and disease-free survival in hepatocellular carcinoma patients [50]. PCAT6 may play an oncogenic role in lung cancer progression, and it negatively correlates with the overall survival of lung cancer patients [51]. MIAT may promote the development of lung adenocarcinoma via the MIAT-miR-106-MAPK signalling pathway loop [52]. Oestrogen increases HOTAIR levels via GPER-mediated miR-148a inhibition and is an independent prognostic marker of metastasis in breast cancer [53, 54]. Moreover, increased expression of DSCAM-AS1 was associated with a worse overall survival in breast cancer patients according to our in silico analysis, and knockdown of DSCAM-AS1 inhibited breast cancer cell proliferation, increased apoptosis and inhibited cell cycle progression in this study. We obtained similar results in a previous report [55]. Antisense lncRNAs can regulate their corresponding sense mRNAs at different levels, including transcriptional interference, imprinting, alternative splicing, translation or RNA editing [56]. However, the sense mRNA DSCAM was not regulated by DSCAM-AS1 [55]. Thus, trans-regulation may be associated with the role of DSCAM-AS1 in breast cancer. Regarding DLEU2 and TINCR, it appears to be controversial that DLEU2 and TINCR would act differently in various malignancies. DLEU2 inhibits cell proliferation and tumour progression through the regulation of miR-15a/miR-16-1 in chronic lymphocytic leukaemia [57, 58]. However, our in silico analysis suggest that, in breast cancer, it may be oncogenic, in contrast to its reported role as a tumour suppressor in chronic lymphocytic leukaemia. For TINCR, this lncRNA is induced by SP1 and promotes cell proliferation in gastric cancer and oesophageal squamous cell carcinoma, a finding that is in accordance with our results in breast cancer [59, 60]. However, in colorectal cancer, the loss of TINCR expression promotes proliferation and metastasis by activating EpCAM cleavage [61]. The functional diversity of DLEU2 and TINCR in various malignancies may explain this discrepancy, and subsequent research is necessary to reveal the potential roles of these two lncRNAs.

The identification of these oncogenic lncRNAs in this study may add vital significance for clinical practice. The study addresses three issues that are not present in other studies. First, a panel of oncogenic lncRNAs was identified via genome-wide in silico analysis. It provides a more efficient step in research methodology and expands the scope of studied lncRNAs that are associated with patient outcome. Hence, the drawbacks of a single lncRNA study model were avoided. Second, the breast cancer prognosis-associated lncRNAs identified via data integration and analysis are definite in this study, and they can be applied for the evaluation of cancer characteristics and patient survival potential. In this way, clinical examination of HOTAIR, LINC00115, MCM3AP-AS1, TINCR, PPP1R26-AS1, and DSCAM-AS1 may be beneficial for comprehensive management. Third, breast cancer subtype-specific lncRNAs were also obtained via high-throughput sequencing. Such oncogenic lncRNAs should be not underestimated for their clinical significance. From a clinical viewpoint, owing to the varying objectivity of pathologists and different qualities of antibodies to ER, PR, HER-2 and Ki-67 used in immunohistochemical methods, determining the molecular subtype for an individual’s breast cancer may be influenced by subjective and objective factors. Many obstacles, including economic and technical factors, must be overcome to achieve the standardization of immunohistochemical methods among laboratories and greater accessibility of quality assurance programmes. From our sequencing results and TCGA data, TINCR, LINC00511, and PPP1R26-AS1 were identified and were found to represent HER-2, triple-negative and luminal B subtypes of breast cancer, respectively. Thus, examination of these BCSPLs may complement the use of the aforementioned parameters, and this approach may decrease the misdiagnosis rate and optimize costs in clinical practice.

We acknowledge several limitations of our study. First, not all the datasets that include lncRNA expression and follow-up information in the GEO could be accessed. Thus, the oncogenic lncRNA landscape of breast cancer indicated in this study would not be sufficiently comprehensive. Second, the biological functions and detailed mechanisms of these oncogenic lncRNAs were not elucidated. Additionally, further in-depth efforts will be needed to investigate other oncogenic lncRNAs besides TINCR, HOTAIR and DSCAM-AS1 with large sample sizes and a focus on molecular mechanisms.

## Conclusions

Taken together, these findings broaden the oncogenic lncRNA landscape of breast cancer and provide insights into the roles of these oncogenic lncRNAs. The results may aid in the comprehensive management of breast cancer.

## Additional files

Additional file 1: Table S1. The clinicopathological characteristics of the patients. (XLS 19 kb)

Additional file 2: Table S2. Differentially expressed genes in Cohort I. (XLS 96 kb)

Additional file 3: Table S3. Differentially expressed genes in Cohort II. (XLS 8892 kb)

Additional file 4: Table S4. Gene ontology term enrichment (GO) and KEGG pathway analysis for upregulated lncRNAs. (XLS 326 kb)

Additional file 5: Table S5. GOTERM_CC_DIRECT and GOTERM_MF_DIRECT for upregulated lncRNAs. (XLSX 41 kb)

Additional file 6: Table S6. Gene ontology term enrichment (GO) and KEGG pathway analysis for differentially expressed mRNAs. (XLS 909 kb)

Additional file 7: Table S7. Differentially expressed genes of RNA-seq in our Cohort. (XLS 2231 kb)

Additional file 8: Table S8. Gene ontology term enrichment (GO) and KEGG pathway analysis for linc00511 in TCGA. (XLSX 21 kb)

Additional file 9: Figure S1. LINC00511 expression was high in patients with BRCA1 or RB1 or TP53 mutation than those with wild type. (PDF 116 kb)

Additional file 10: Table S9. StarBase v2.0 database prediction for the relationship betwwen LINC00511 and tumour suppressor microRNAs. (XLSX 9 kb)

Additional file 11: Table S10. 26 datasets with 4140 breast cancer patients for identifying prognosis-associated lncRNAs. (XLS 24 kb)

Additional file 12: Table S11. DNA methylation and transcription factors regulation for lncRNAs. (XLS 27 kb)

Additional file 13: Figure S2. TINCR mediates the tumourigenesis of breast cancer partly via regulation of apoptosis pathway and TINCR-MiR-125-ERBB2 axis in UACC-812 cell line. (PDF 1571 kb)

Additional file 14: Table S12. TINCR is negatively expressed with several genes involved in apoptosis pathway via analysis of TCGA breast cancer data. (XLSX 657 kb)

Additional file 15: Figure S3. Oncogenic functions of DSCAM-AS1 and HOTAIR. (PDF 462 kb)

## Abbreviations

BCPALs: Breast cancer prognosis-associated lncRNAs
BCSPLs: Breast cancer subtype-specific lncRNAs
CV: Coefficient of variation
ENCODE: Encyclopedia of DNA Elements
FDR: False discovery rate
GEO: Gene expression omnibus
GO: Gene ontology term enrichment
KEGG: Kyoto encyclopedia of genes and genomes
LncRNA: Long noncoding RNAs
piRNA: Piwi-interacting RNA
siRNA: Small interfering RNA
TCGA: The cancer genome atlas
TUNEL: Transferase mediated deoxyuridine triphosphate-digoxigenin nick end-labelling assay
