# Supplementary material for: Oncogenic long noncoding RNA landscape in breast cancer
Source: Mol Cancer. 2017 Jul 24;16:129. doi: 10.1186/s12943-017-0696-6 (PMC5525255; doi:10.1186/s12943-017-0696-6)
Supplement: Supplementary file 9 — LINC00511 expression was high in patients with BRCA1 or RB1 or TP53 mutation than those with wild type. (PDF 116 kb) [file 12943_2017_696_MOESM9_ESM.pdf]

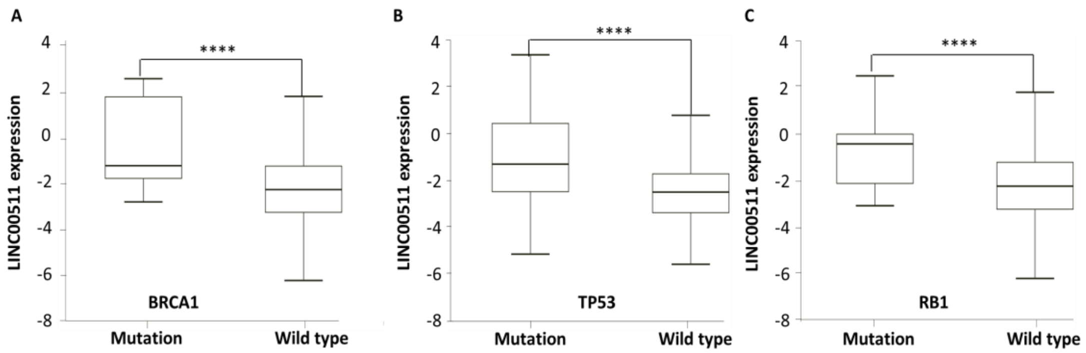

Figure S1. LINC00511 expression in patients with mutation in TCGA.

A) LINC00511 expression was higher in patients with BRCA1 mutation than those with wild type.

B) LINC00511 expression was higher in patients with TP53mutation than those with wild type.

C) LINC00511 expression was higher in patients RB1 mutation than those with wild type.

**** P < 0. 0001
