# Supplementary material for: Oncogenic long noncoding RNA landscape in breast cancer
Source: Mol Cancer. 2017 Jul 24;16:129. doi: 10.1186/s12943-017-0696-6 (PMC5525255; doi:10.1186/s12943-017-0696-6)
Supplement: Supplementary file 15 — Oncogenic functions of DSCAM-AS1 and HOTAIR. (PDF 462 kb) [file 12943_2017_696_MOESM15_ESM.pdf]

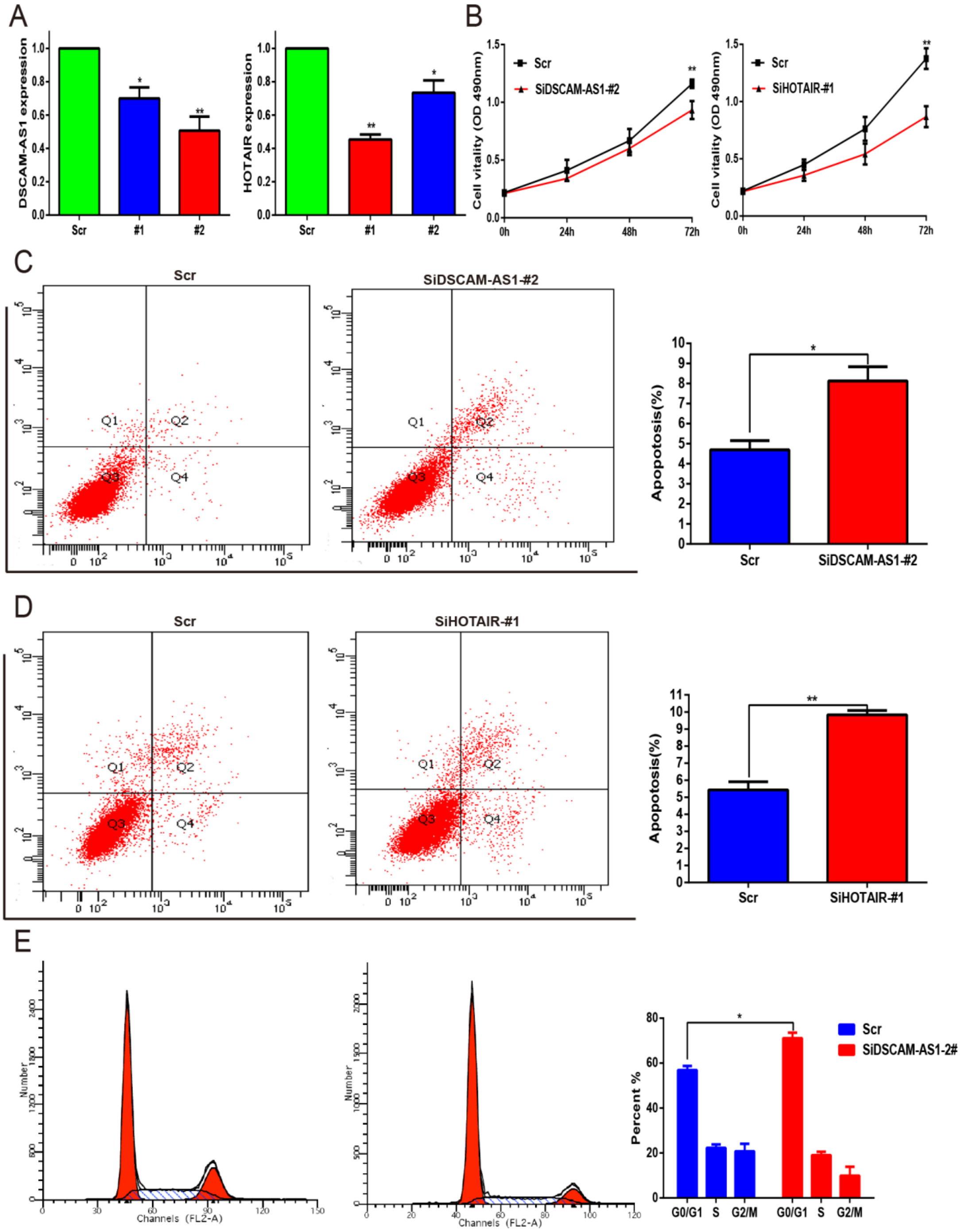

Figure S2. Oncogenic functions of DSCAM-AS1 and HOTAIR. A) Knockdown efficiency of siRNAs targeting DSCAM-AS1 and HOTAIR, as determined by qRT-PCR. Quantitative normalization of HOTAIR and DSCAM-AS1 was performed in each sample by using GAPDH expression as an internal control. The relative levels of HOTAIR and DSCAM-AS1 vs. GAPDH were determined by the comparative CT ($2^{-\Delta\Delta CT}$) method. B) The CCK-8 assay was conducted to measure MCF7 and T47D cell proliferation after knockdown of DSCAM-AS1 and HOTAIR, respectively. C-D: Apoptosis analysis of MCF7(C) and T47D (D) cells after knockdown of DSCAM-AS1 and HOTAIR, respectively, via flow cytometry. E) Determination of cell cycle distribution of MCF7 cells after knockdown of DSCAM-AS1 via flow cytometry. ** P < 0. 01; * P < 0.05. Each assay was performed in triplicate.
