# Supplementary material for: Oncogenic long noncoding RNA landscape in breast cancer
Source: Mol Cancer. 2017 Jul 24;16:129. doi: 10.1186/s12943-017-0696-6 (PMC5525255; doi:10.1186/s12943-017-0696-6)
Supplement: Supplementary file 13 — TINCR mediates the tumourigenesis of breast cancer partly via regulation of apoptosis pathway and TINCR-MiR-125-ERBB2 axis in UACC-812 cell line. (PDF 1571 kb) [file 12943_2017_696_MOESM13_ESM.pdf]

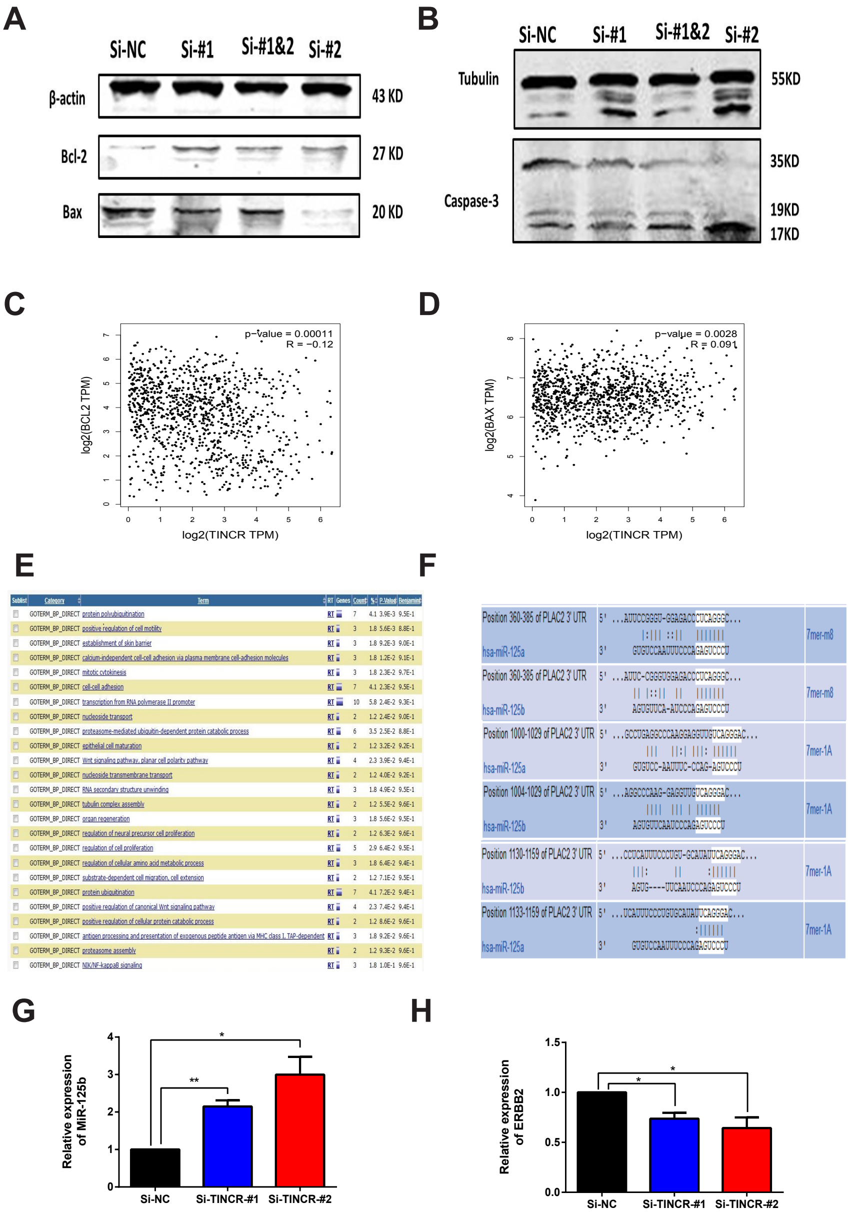

Sublist	Category	Term	RT	Genes	Count	%	P-Value	Benjamini
	GOTERM_BP_DIRECT	protein polyubiquitination	RT		7	4.1	3.9E-3	9.5E-1
	GOTERM_BP_DIRECT	positive regulation of cell motility	RT		3	1.8	5.6E-3	8.8E-1
	GOTERM_BP_DIRECT	establishment of skin barrier	RT		3	1.8	9.2E-3	9.0E-1
	GOTERM_BP_DIRECT	calcium-independent cell-cell adhesion via plasma membrane cell-adhesion molecules	RT		3	1.8	1.2E-2	9.1E-1
	GOTERM_BP_DIRECT	mitotic cytokinesis	RT		3	1.8	2.3E-2	9.7E-1
	GOTERM_BP_DIRECT	cell-cell adhesion	RT		7	4.1	2.3E-2	9.5E-1
	GOTERM_BP_DIRECT	transcription from RNA polymerase II promoter	RT		10	5.8	2.4E-2	9.3E-1
	GOTERM_BP_DIRECT	nucleoside transport	RT		2	1.2	2.4E-2	9.0E-1
	GOTERM_BP_DIRECT	proteasome-mediated ubiquitin-dependent protein catabolic process	RT		6	3.5	2.5E-2	8.8E-1
	GOTERM_BP_DIRECT	epithelial cell maturation	RT		2	1.2	3.2E-2	9.2E-1
	GOTERM_BP_DIRECT	Wnt signaling pathway, planar cell polarity pathway	RT		4	2.3	3.9E-2	9.4E-1
	GOTERM_BP_DIRECT	nucleoside transmembrane transport	RT		2	1.2	4.0E-2	9.2E-1
	GOTERM_BP_DIRECT	RNA secondary structure unwinding	RT		3	1.8	4.9E-2	9.5E-1
	GOTERM_BP_DIRECT	tubulin complex assembly	RT		2	1.2	5.5E-2	9.6E-1
	GOTERM_BP_DIRECT	organ regeneration	RT		3	1.8	5.6E-2	9.5E-1
	GOTERM_BP_DIRECT	regulation of neural precursor cell proliferation	RT		2	1.2	6.3E-2	9.6E-1
	GOTERM_BP_DIRECT	regulation of cell proliferation	RT		5	2.9	6.4E-2	9.5E-1
	GOTERM_BP_DIRECT	regulation of cellular amino acid metabolic process	RT		3	1.8	6.4E-2	9.4E-1
	GOTERM_BP_DIRECT	substrate-dependent cell migration, cell extension	RT		2	1.2	7.1E-2	9.5E-1
	GOTERM_BP_DIRECT	protein ubiquitination	RT		7	4.1	7.2E-2	9.4E-1
	GOTERM_BP_DIRECT	positive regulation of canonical Wnt signaling pathway	RT		4	2.3	7.4E-2	9.4E-1
	GOTERM_BP_DIRECT	positive regulation of cellular protein catabolic process	RT		2	1.2	8.6E-2	9.6E-1
	GOTERM_BP_DIRECT	antigen processing and presentation of exogenous peptide antigen via MHC class I, TAP-dependent	RT		3	1.8	9.2E-2	9.6E-1
	GOTERM_BP_DIRECT	proteasome assembly	RT		2	1.2	9.3E-2	9.6E-1
	GOTERM_BP_DIRECT	NIK/NF-kappaB signaling	RT		3	1.8	1.0E-1	9.6E-1
